# Human Infection with *Sporolactobacillus laevolacticus*, Marseille, France

**DOI:** 10.3201/eid2111.151197

**Published:** 2015-11

**Authors:** Cédric Abat, Jad Kerbaj, Gregory Dubourg, Vincent Garcia, Jean-Marc Rolain

**Affiliations:** Aix-Marseille Université, Marseille, France

**Keywords:** Sporolactobacillus laevolacticus, bacteria, human infection, blood, cellulitis, Marseille, France

**To the Editor:** *Sporolactobacillus laevolacticus,* formerly known as *Bacillus laevolacticus*, is a gram-positive, acid-tolerant, catalase-positive, facultatively anaerobic and mesophilic bacteria initially isolated from the rhizosphere of wild plants (*1*,*2*). However, there have been no reports of its isolation from humans. We report *S. laevolacticus* associated with a wound infection and cellulitis in a patient hospitalized in Marseille, France.

In March 2015, a 47-year-old man with no underlying disease was admitted to the emergency unit of the North Hospital in Marseille, France. He had an infected wound on his right foot that occurred after he jogged barefoot during a vacation in Comoros, but the patient did not know how he obtained the wound and had not taken any antiinflammatory drugs. The foot became swollen, red, hot, and painful. He visited a doctor during his vacation and was prescribed antiinflammatory drugs and antimicrobial drugs, including a second-generation cephalosporin and ofloxacin.

The patient returned to Marseille, but the infection persisted. At admission, the patient was apyretic but had high levels of C-reactive protein (85.7 mg/L [reference range 1–3 mg/L]) and fibrinogen (8.35 g/L), which indicated inflammation. His leukocyte count was normal (9.29 ×10^9^ cells/L) but his procalcitonin level (0.19 µg/L) was increased, which suggested that the infection had not been cured. A cellulitis abscess was suspected, and surgical cleaning and drainage was performed on March 10 (Figure, panels A, B).

**Figure:**
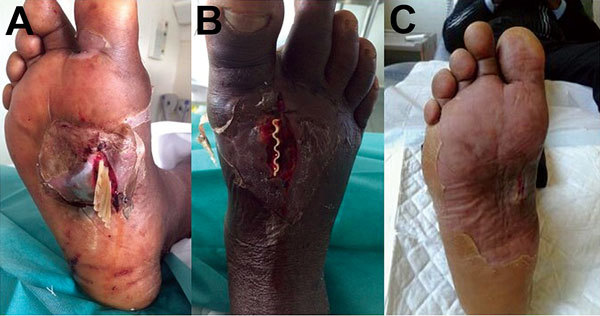
A) Foot of a 47-year-old man showing wound infected with *Sporolactobacillus laevolaticus*, Marseille, France. B) Drainage of a cellulitis abscess. C) Extent to which the wound on the arch of the foot had healed 6 weeks after surgery and antimicrobial drug therapy. A color version of this figure is available online (http://wwwnc.cdc.gov/EID/article/21/11/15-1197-F1.htm).

Samples were collected during surgery and probabilistic antimicrobial drug therapy, including tazocillin, clindamycin, and vancomycin, was initiated. Abscess puncture liquid collected during surgery was sterile when incubated directly on Columbia and Polyvitex agar plates (bioMérieux, Craponne, France). However, a surgical sample inoculated into a blood culture bottle grew gram-positive bacilli after 4 days.

Subculture colonies were identified by using matrix-assisted laser desorption/ionization time of flight (MALDI-TOF) mass spectrometry (Bruker, Leipzig, Germany) as *S. laevolaticus* (score 1.88). Identification was confirmed by PCR amplification of the 16S RNA gene (*3*). A 944-bp sequence showed 99.5% similarity with that of a known *S. laevolaticus* strain (GenBank accession no. AB362648) by BLAST analysis (http://www.ncbi.nlm.nih.gov).

The *S. laevolaticus* strain was susceptible to amoxicillin, amoxicillin/clavulanate, imipenem, metronidazole, clindamycin, and vancomycin. The antimicrobial drug regimen was then changed to clindamycin and trimethoprim/sulfamethoxazole, and the patient showed an excellent clinical outcome. The patient was considered clinically cured 7 weeks later (Figure, panel C).

*S. laevolacticus* has been studied for its capacity to survive extreme conditions and for its fermentation system (*4*–*8*). The fact that the bacterium has not been previously isolated from humans might be because it was isolated only from plant rhizospheres (*2*), so human studies have not been conducted. In addition, conventional identification methods, such as the VITEK 2 system (bioMérieux) or the API system (bioMérieux), cannot identify *S. laevolacticus*. Since September 2009, we have used MALDI-TOF mass spectrometry in North Hospital for routine identification of bacterial species isolated from clinical samples (*9*). This strategy increases our capacity to detect rare bacterial species, including emerging pathogens (*10*).

The bacterial species was accurately identified by using MALDI-TOF and then confirmed by using a 16S RNA PCR. Because the bacterium was originally isolated from a plant rhizosphere and the patient was hospitalized with an open wound in the foot and bacteremia, we speculate that the infection was the direct result of close extended contact between the wound and soil infected with the bacteria. This case confirms that *S. laevolaticus* can be responsible for human infections and suggests that this bacterial species could be an emerging opportunistic pathogen responsible for human infections.
